# Donor IFNL4 Genotype Is Associated with Early Post-Transplant Fibrosis in Recipients with Hepatitis C

**DOI:** 10.1371/journal.pone.0166998

**Published:** 2016-11-22

**Authors:** Taylor Aiken, Ari Garber, Dawn Thomas, Nicole Hamon, Rocio Lopez, Rajesh Konjeti, Arthur McCullough, Nizar Zein, John Fung, Medhat Askar, Binu V. John

**Affiliations:** 1 Cleveland Clinic Lerner College of Medicine, Cleveland Clinic, Cleveland, Ohio, United States of America; 2 Digestive Diseases Institute, Cleveland Clinic, Cleveland, Ohio, United States of America; 3 Allogen Laboratories, Cleveland Clinic, Cleveland, Ohio, United States of America; 4 Quantitative Health Sciences, Cleveland Clinic, Cleveland, Ohio, United States of America; 5 Virginia Commonwealth University School of Medicine, Richmond, Virginia, United States of America; 6 Transplant Immunology, Baylor University Medical Center, Dallas, Texas, United States of America; 7 Gastroenterology and Hepatology, Hunter Holmes McGuire VA Medical Center, Richmond, Virginia, United States of America; Westmead Millennium Institute for Medical Research, AUSTRALIA

## Abstract

**Background and Aims:**

Early post-transplant hepatic fibrosis is associated with poor outcomes and may be influenced by donor/recipient genetic factors. The rs368234815 IFNL4 polymorphism is related to the previously described IL28B polymorphism, which predicts etiology-independent hepatic fibrosis. The aim of this study was to identify the impact of donor and/or recipient IFNL4 genotype on early fibrosis among patients transplanted for hepatitis C (HCV).

**Methods:**

Clinical data were collected for 302 consecutive patients transplanted for HCV. 116 patients who had available liver biopsies and donor/recipient DNA were included. 28% of these patients with stage 2 fibrosis or greater were compared to patients without significant post-transplant fibrosis with respect to clinical features as well as donor/recipient IFNL4 genotype.

**Results:**

The IFNL4 TT/TT genotype was found in 26.0% of recipients and 38.6% of donors. Patients who developed early post-transplant fibrosis had a 3.45 adjusted odds of having donor IFNL4 TT/TT genotype (p = 0.012). Donor IFNL4 TT/TT genotype also predicted decreased overall survival compared to non-TT/TT genotypes (p = 0.016).

**Conclusions:**

Donor IFNL4 TT/TT genotype, a favorable predictor of spontaneous HCV clearance pre-transplant, is associated with increased early post-transplant fibrosis and decreased survival.

## Introduction

End-stage liver disease attributable to chronic HCV infection remains a leading indication for liver transplantation [1]. Some patients develop hepatic fibrosis early after transplant, and this may contribute to graft failure in a subset of patients. The degree of post-transplant fibrosis varies from person to person and genetic factors play an important role in disease progression [2]. A single nucleotide polymorphism (rs12979860) upstream of the interferon lambda 3 gene (IFNL3, IL28B) has been shown to predict post-transplant fibrosis and fibrosing cholestatic hepatitis in the absence of treatment [3,4]. While the IL28B C/C genotype is associated with increased spontaneous clearance of HCV and favorable treatment response, patients who receive a liver with the C/C genotype in the transplant setting have higher rates of histologic recurrence and fibrosis [5].

Subsequent studies have identified a novel dinucleotide polymorphism (ss469415590 also called rs368234815, TT/ΔG) that is strongly associated with spontaneous HCV clearance and is found in strong linkage disequilibrium with rs12979860 [6]. The dinucleotide variant is located 367bp upstream of rs12979860 and is comprised of a one-base deletion polymorphism (loss of T) and a one-base substitution variant (T>G) [6]. The TT/TT genotype is associated with increased spontaneous HCV clearance relative to the ΔG/ΔG genotype [6]. The identification of the rs368234815 polymorphism is particularly interesting as it suggests a possible molecular mechanism underlying its predictive value for viral clearance. The ΔG frame-shift variant creates a novel gene, IFNL4, which is transiently activated in response to HCV infection [6]. IFNL4 encodes a protein which induces phosphorylation and activation of STAT1 and STAT2, activating downstream interferon-stimulated response elements (ISRE). This leads to activation of interferon-stimulated genes (ISGs) in a manner similar to type I and type III interferon signaling, generating an antiviral response in hepatocytes [6].

Studies have shown that IFNL4 polymorphisms are a predictor of treatment response with interferon-based regimens with genotype 2 and 3 as well as for simeprevir and interferon-based therapy for genotype 1 [7–9]. In addition, polymorphisms in both IFNL3 and IFNL4 have been shown to be associated with high serum and hepatic iron concentrations [10]. Though there is only a weak association between response to direct active anti-viral therapy (DAA) and IFNL4, the presence of the beneficial IFNL4 polymorphisms were associated with higher prevalence of the NS5A Y93H resistance associated variant (RAV) [11,12]. In the context of liver transplantation, IFNL4 polymorphisms have been shown to be associated with sustained viral response (SVR) to interferon-based regimens, with recipient IFNL4 TT/TT genotype being associated with higher SVR rates [13].

The impact of IFNL4 polymorphisms on hepatic fibrosis has not been studied in the context of transplant. The aim of this study was to study the impact of donor and recipient rs368234815 genotype on early post-transplant fibrosis in treatment naïve patients. Based on observations in IL28B, we hypothesized that donor rs368234815 TT/TT genotype, a predictor of spontaneous viral clearance in the pre-transplant setting, is associated increased inflammation and early post-transplant fibrosis.

## Patients and Methods

### Patients

We collected clinical data for 302 consecutive patients who underwent liver transplant at Cleveland Clinic (Cleveland, OH) for chronic HCV from January 1, 2006 to December 31, 2011. We chose patients prior to the routine use of interferon free direct anti-viral treatments as these patients underwent routine surveillance protocol liver biopsies. Patients who did not have available donor/recipient DNA or liver biopsy between 6–18 months were excluded. All patients were followed up according to a standard protocol. The study protocol conformed to the ethical guidelines of the 1975 Declaration of Helsinki and 2008 Declaration of Istanbul and was approved by the Institutional Review Board at Cleveland Clinic. Written consent for the storage of tissue specimens and clinical data for research purposes was obtained for each patient in accordance with the Institutional Review Board at Cleveland Clinic. None of the transplant donors were from a vulnerable population and all donors or next of kin provided written informed consent that was freely given.

Patients underwent protocol liver biopsies at 6 months and 1 year post-transplantation and yearly thereafter. Treatment was generally only offered to patients with biopsy-proven fibrosis. As such, few patients underwent treatment prior to first biopsy. Liver biopsies performed between 6–18 months post-transplant were graded and staged according to Batts and Ludwig criteria [14]. A previously accepted cutoff of Batts and Ludwig stage 2–4 was defined as significant fibrosis [15]. Donor and recipient genotypes were compared among patients who developed significant fibrosis on any protocol biopsy between 6–18 months and patients with mild or no fibrosis. Additional clinical variables were obtained from a prospectively maintained database of patients receiving liver transplants at Cleveland Clinic. HCV genotyping was performed using the line probe assay in all patients.

### DNA Collection and Genotyping

Donor and recipient DNA was extracted from whole blood obtained and stored at the time of transplant according to standard protocol. The rs12979860 polymorphism was analyzed by TaqMan SNP genotyping assay (Life Technologies, Carlsbad, CA). The C allele was detected using a VIC-labeled probe with the sequence TGGTTCGCGCCTTC and T allele was detected using a FAM-labeled probe with the sequence CTGGTTCACGCCTTC [6]. The rs368234815 polymorphism was analyzed in the same manner. The TT allele was detected using a VIC-labeled probe with the sequence ATCGCAGAAGGCC and ΔG allele was detected using a FAM-labeled probe with the sequence ATCGCAGCGGCCC [6].

### Statistical Analysis

Data are presented as mean ± standard deviation, median [25^th^, 75^th^ percentiles] or N (%). The prevalence of ss469425590 genotypes among donor and recipients was estimated by calculating the percentage of patients with ΔG/ΔG, TT/ΔG and TT/TT. A univariable analysis was performed to assess factors associated with developing fibrosis stage 2–4 post-transplant; Pearson’s chi-square tests were used for categorical variables and analysis of variance (ANOVA) or the non-parametric Kruskal-Wallis tests were used for continuous or ordinal factors. In addition, multivariable logistic regression was performed to assess association between genotype and presence of moderate fibrosis after adjusting for potential confounders; fibrosis 2–4 was modeled as the outcome with ss469424590 genotype and other clinical characteristics as the independent variables. Survival analysis was also performed. Follow-up time was defined as months from OLT to death and subjects who remained alive were censored on 7/1/2013. Kaplan-Meier plots were constructed and log-rank tests were used to compare the genotypes. A p<0.05 was considered statistically significant. SAS (version 9.3, The SAS Institute, Cary, NC) and R (version 3.0.3, The R Foundation for Statistical Computing, Vienna, Austria) were used to perform all analyses.

## Results

### Patient Characteristics

**Fig 1:**
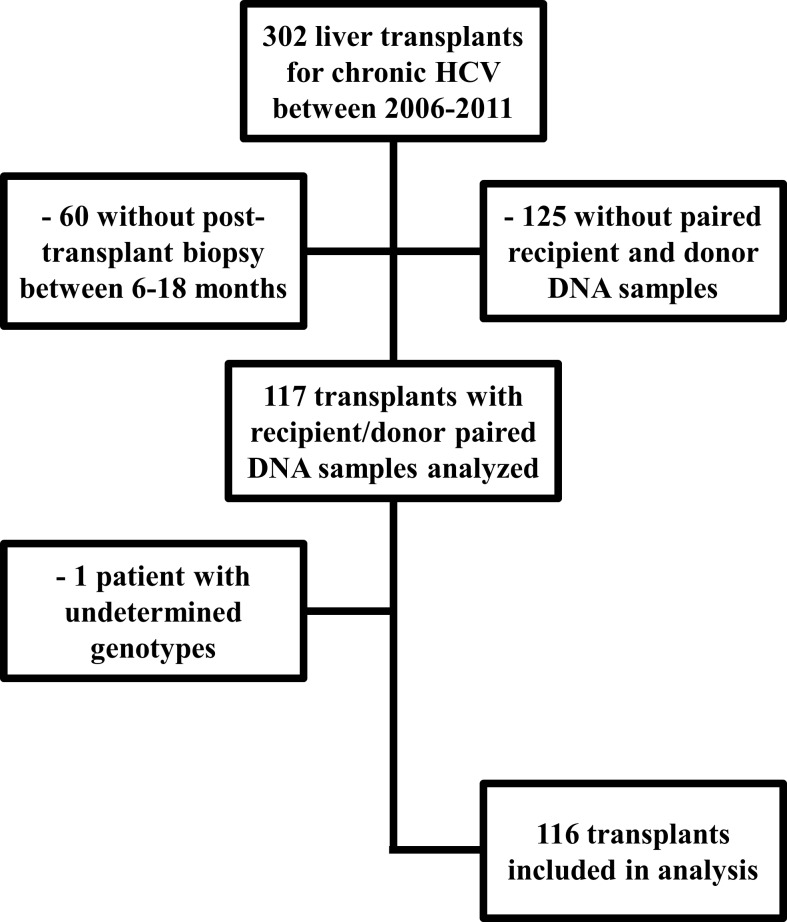
Identification of Patient Population.

Clinical data were collected for a total of 302 consecutive patients who were transplanted for HCV during the study period. 116 patients who had available donor DNA, recipient DNA, and liver biopsy between 6–18 months were included in the analysis (Fig 1). The study population was similar to the overall transplant recipient population for HCV at Cleveland Clinic, 74% were males and 19.3% were African Americans. 33 patients developed significant fibrosis on liver biopsy (28%), which is consistent with previous reports [5]. The mean time from transplant to biopsy was 10.7 ± 2.4 months; there was no difference in follow-up time between those who progressed to stage 2–4 and those who did not (p = 0.90).

Table 1 shows the donor and recipient characteristics for patients with and without significant fibrosis. Patients with significant fibrosis were more likely to be African American, which is consistent with previously observed racial variation in HCV progression. There was no significant difference in recipient age, recipient gender, HCV genotype, donor age, cold ischemia time, or donor risk index (DRI) among the 2 groups.

**Table 1:** Patient Characteristics.

Factor	Fibrosis 2-4(N = 33)	Fibrosis 0–1 (N = 83)	p-value
Recipient Age at Transplant	54.9±6.6	56.2±6.3	0.33^a^
Recipient Male Sex	24 (72.7%)	62 (74.7%)	0.83^c^
Recipient African-American	13 (40.6%)	8(10.4%)	0.001^c^
Recipient CMV+	25 (75.8%)	59 (71.1%)	0.61^c^
Diagnosis			0.090^c^
. HCV	11 (33.3%)	19 (22.9%)	
. HCV + alcohol	7 (21.2%)	14 (16.9%)	
. HCV + HCC	11 (33.3%)	42 (50.6%)	
. HCV + alcohol + HCC	1 (3.0%)	7 (8.4%)	
. HCV + Other	3 (9.1%)	1 (1.2%)	
HCV Genotype			0.12^c^
. 1	29 (87.9%)	50 (65.8%)	
. 2	1 (3.0%)	7 (9.2%)	
. 3	2 (6.1%)	15 (19.7%)	
. 4	1 (3.0%)	4 (5.3)	
Donor Age at Death	39.5±15.2	40.5±13.8	0.73^a^
Cold-Ischemia Time	7.6±1.7	7.2±1.7	0.38^a^
Donor Risk Index	2.1±0.51	2.1±0.49	0.68^a^

Values presented as Mean ± SD or N (%).

p-values: a = ANOVA, c = Pearson's chi-square test.

### Distribution of Genotypes

The distribution of genotypes for the rs368234815 and rs12979860 polymorphisms among 116 included patients is shown in Table 2. In 2 patients the recipient rs368234815 genotype could not be determined and in 12 patients the donor rs368234815 genotype could not be determined. There were no significant differences between groups for unadjusted comparison. The linked rs368234815 and rs12979860 polymorphisms demonstrated similar allele distributions for the donor and recipient population.

**Table 2:** Allele Frequency for Patient Population.

	Overall		Fibrosis 2–4		Fibrosis 0–1		
	(N = 116)		(N = 33)		(N = 83)		
Factor	n		n		n		p-value
Donor rs368234815	114		32		82		0.094
. TT/TT		44 (38.6%)		17 (53.1%)		27 (32.9%)	* *
. TT/ΔG		49 (43.0%)		12 (37.5%)		37 (45.1%)	* *
. ΔG/ΔG		21 (18.4%)		3 (9.4%)		18 (22.0%)	* *
Recipient rs368234815	104		31		73		0.07
. TT/TT		27 (26.0%)		4 (12.9%)		23 (31.5%)	* *
. TT/ΔG		52 (50.0%)		16 (51.6%)		36 (49.3%)	* *
. ΔG/ΔG		25 (24.0%)		11 (35.5%)		14 (19.2%)	* *
Donor rs12979860	116		33		83		0.26
. C/C		48 (41.4%)		17 (51.5%)		31 (37.3%)	* *
. C/T		49 (42.2%)		13 (39.4%)		36 (43.4%)	* *
. T/T		19 (16.4%)		3 (9.1%)	** **	16 (19.3%)	* *
Recipient rs12979860	116		33		83		0.25
. C/C		30 (25.9%)		5 (15.2%)		25 (30.1%)	* *
. C/T		61(52.6%)		20 (60.6%)		41 (49.4%)	* *
. T/T		25 (21.6%)		8 (24.2%)		17 (20.5%)	* *

Values presented as N (column %) with Pearson’s chi-square test.

### rs368234815 Genotype and Fibrosis

The rs368234815 TT/TT genotype was found in 38.6% of donors and 26.0% of recipients. On univariable analysis, recipient race [African American] and donor TT/TT were found to be significantly associated with fibrosis. The donor or recipient rs12979860 (IL28B) polymorphism was not a significant predictor of early post-transplant fibrosis on univariable analysis. On multivariable analysis adjusting for African American race and pre-transplant HCC, donor TT/TT genotype was associated with a 3.45 odds of developing fibrosis (Table 3). No other comparisons were found to be significant.

**Table 3:** Adjusted Comparison of Genotypes.

Genotype	Unadjusted OR	p-value	Adjusted* OR	p-value
Donor rs368234815: TT/TT vs non-TT/TT	2.33 (1.01, 5.26)	0.049	3.45 (1.32, 9.09)	0.012
Recipient rs368234815: TT/TT vs non-TT/TT	0.32 (0.10, 1.03)	0.056	0.53 (0.15, 1.82)	0.3
Donor rs12979860: CC vs non-CC	1.79 (0.77, 4.00)	0.16	2.38 (0.94, 6.25)	0.067
Recipient rs12979860: CC vs non-CC	0.42 (0.14, 1.19)	0.10	0.45 (0.14, 1.47)	0.19

* Recipient and donor genotypes are adjusted for recipient African-American vs. non-African-American race and recipient diagnosis (HCC vs no-HCC).

Patients who developed significant fibrosis had a higher proportion of African Americans. Therefore, we also analyzed the impact of the two polymorphisms in the African American recipient population of our cohort. In this cohort, the donor TT/TT genotype trended towards a higher odds (6.8) of developing fibrosis but the comparisons were not statistically significant due to relatively low numbers (p = 0.085).

### Survival Analysis

**Fig 2:**
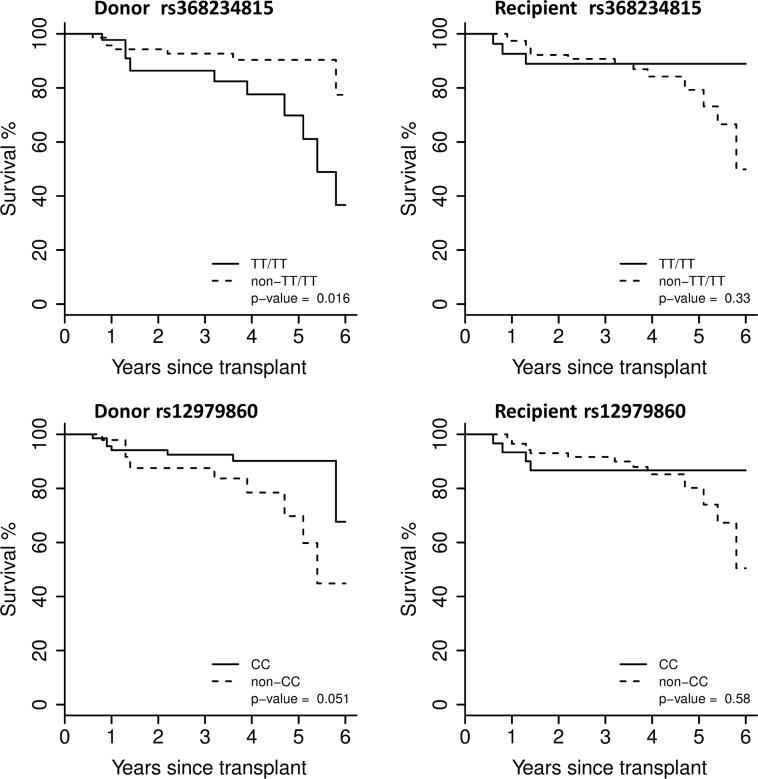
Kaplan-Meier curves for donor/recipient rs368234815 genotype and donor/recipeient rs12979860 genotype.

The Kaplan-Meier plots for post-transplant survival for rs368234815 genotype and rs12979860 genotype are presented in Fig 2. Subjects with a donor that had rs368234815 TT/TT genotype had significantly worse survival than those with rs368234815 non-TT/TT donors (p = 0.016). There was no significant difference for recipient rs368234815 genotype or donor/recipient rs12979860 genotype. When survival was compared for donor/recipient rs36823481 combinations, a trend toward shorter survival was observed in non-TT/TT recipients who received a liver from a donor with TT/TT genotype (S1 Fig).

## Discussion

We evaluated the potential association between rs368234815 genotype and post-transplant fibrosis in patients who underwent liver transplant for HCV cirrhosis. We show that in our cohort, patients who developed early post-transplant fibrosis had a 3.45 adjusted odds of having donor IFNL4 TT/TT genotype (p = 0.012, Table 3).These findings are consistent with the linked rs12979860 IFNL3 polymorphism (IL28B), where the favorable C/C genotype predicts increased post-transplant fibrosis when acquired in the donor liver [2,5]. However, in regards to post-transplant fibrosis, the donor IFNL4 genotype achieved significance, while recipient and donor IFNL3 genotype did not (Table 3).

The IFNL3 rs12979860 (IL28B) polymorphism was first identified as being predictive of treatment response in the non-transplant setting, with the C/C genotype being associated with a higher probability of achieving SVR to interferon-based regimens [16]. This finding was confirmed in subsequent studies in the transplant setting, with donor C/C genotype being associated with higher SVR rates following transplant [2]. However, the donor C/C genotype was also found to be associated with increased early HCV-related fibrosis following transplant [5]. With the identification of the IFNL4 TT/TT genotype as a potentially stronger predictor of SVR in the non-transplant setting, the present study was conducted to in order to confirm the suspected association of the donor IFNL4 polymorphism with early post-transplant fibrosis [6]. Our finding that donor IFNL4 TT/TT genotype is associated with significant fibrosis is therefore consistent with previous observations with regards to IFNL3. Confirmation of this finding is important as the functional role of IFNL4 is studied further at the basic science level and in the context of HCV-independent hepatic fibrosis.

The functional role of the IFNL4 rs368234815 allows speculation regarding the potential mechanism of increased fibrosis among patients who receive a liver with the TT/TT allele. The TT/TT allele introduces a frame-shift mutation that leads to no expression of IFNL4 [6]. The lack of IFNL4 expression results in decreased expression of ISGs in liver tissues from patients with chronic HCV [17]. It is possible that in patients with donor rs368234815 TT/TT genotype, decreased expression of ISGs is associated with increased progression to fibrosis in the setting of recurrent hepatitis C. Conversely, IFNL3 polymorphisms have been shown to be associated with etiology-independent hepatic fibrosis, suggesting that the mechanism underlying the association between IFNL4 TT/TT genotype and post-transplant fibrosis might be HCV-independent [15]. Further investigation of IFNL4 genotype in non-HCV transplant cohorts might clarify the dependence of the observed association on HCV-recurrence. Finally, it is also possible that the association between the favorable IFNL4 polymorphism and prevalence of resistance associated variants may explain the increased progression to fibrosis [11].

Though we are in the era of near universal cure with currently available direct antiviral regimens, our data remains clinically relevant in the setting of liver transplantation. Many subjects with chronic hepatitis C and decompensated cirrhosis are not treated pre-transplant. This is because successful cure of hepatitis C may place these patients in a so called “liver purgatory” where they remain on the transplant waitlist with a decrease in MELD scores without significant clinical improvement. Pre-transplant cure of hepatitis C may also preclude them from receiving hepatitis C positive grafts. Therefore many transplant centers are waiting until after transplantation to initiate anti-viral treatment. As viremic patients continue to receive transplants and payers refuse coverage for DAA in the early post-transplant period, genetic factors that lead to rapid progression of fibrosis may help to identify patents requiring early therapy. Moreover, in subjects who receive the rs368234815 TT/TT genotype through transplantation, the increased risk of NS5A RAVs may be detrimental.

We recognize several limitations in the present study. Exclusion of patients who did not have donor or recipient DNA or a liver biopsy performed between 6–18 months has the potential of selection bias. However, we separately analyzed baseline characteristics among the study population and the total population of transplanted patients and found no significant differences. Though it would be ideal to perform a genome wide association study, the sample size limited us to explore specific polymorphisms based on our hypothesis regarding specific genetic polymorphisms. The sample size may have also contributed to the lack of a significant association between IFNL3 genotype and fibrosis, a finding that has been observed in multiple larger studies [2,5]. In addition, the enrichment of African Americans among the patients with significant fibrosis may impact the relative distribution of polymorphisms. However, the trends appear to be similar when the African Americans in our cohort were analyzed separately, though numbers limit the ability to adequately interpret sub-groups analysis. Similarly, genotype 1 HCV has been associated with increased fibrosis following liver transplantation and a non-significant trend toward more genotype 1 patients in the fibrosis group was observed in this study [18]. As such, HCV genotype represents an additional potential confounder in addition to race.

Early HCV-related fibrosis following liver transplantation is associated with risk of graft loss. Even in the era of direct acting anti-viral treatment, many patients with decompensated cirrhosis are now being treated post-transplant and donor genetic markers may be a useful tool to predict patients who are at risk to develop early post-transplant fibrosis. More importantly, this data offers insight into the role of novel donor genetic factors in post-transplant fibrosis in HCV.

## Supporting Information

S1 Fig Kaplan-Meier curves for paired donor/recipient rs368234815 genotype. (TIF)
