# Supplementary figures and images for: Donor IFNL4 Genotype Is Associated with Early Post-Transplant Fibrosis in Recipients with Hepatitis C
Source: PLoS One. 2016 Nov 22;11(11):e0166998. doi: 10.1371/journal.pone.0166998 (PMC5119817; doi:10.1371/journal.pone.0166998)

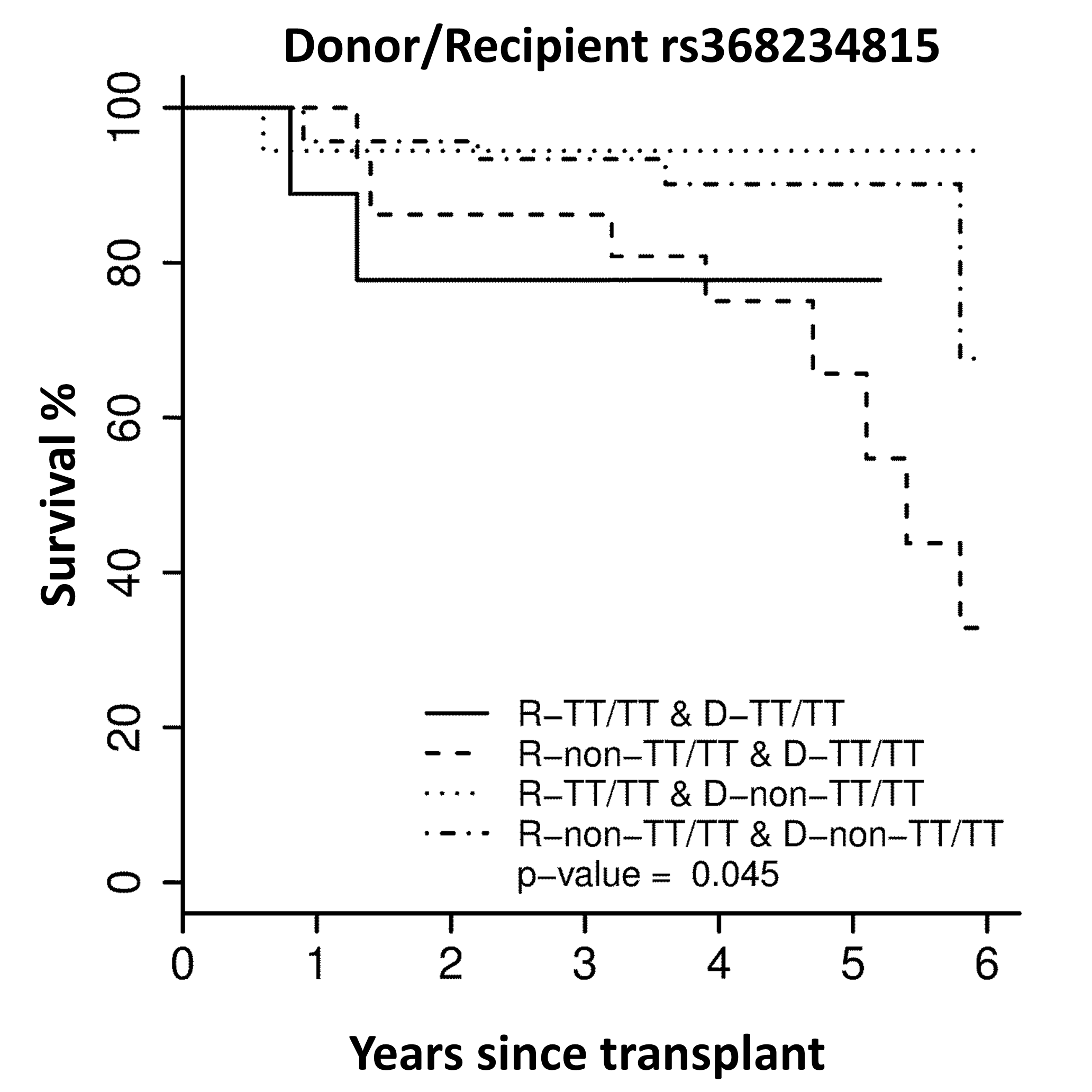

Supplement: S1 Fig — (TIF) [file pone.0166998.s001.tif]
